# Hooked on heart regeneration: the zebrafish guide to recovery

**DOI:** 10.1093/cvr/cvab214

**Published:** 2021-06-23

**Authors:** Katherine M Ross Stewart, Sophie L Walker, Andrew H Baker, Paul R Riley, Mairi Brittan

**Affiliations:** Centre for Cardiovascular Science, University of Edinburgh, The Queen's Medical Research Institute, 47 Little France Crescent, Edinburgh EH16 4TJ, UK; Centre for Cardiovascular Science, University of Edinburgh, The Queen's Medical Research Institute, 47 Little France Crescent, Edinburgh EH16 4TJ, UK; Centre for Cardiovascular Science, University of Edinburgh, The Queen's Medical Research Institute, 47 Little France Crescent, Edinburgh EH16 4TJ, UK; Department of Physiology, Anatomy & Genetics, University of Oxford, Sherrington Building, Sherrington Rd, Oxford OX1 3PT, UK; Centre for Cardiovascular Science, University of Edinburgh, The Queen's Medical Research Institute, 47 Little France Crescent, Edinburgh EH16 4TJ, UK

**Keywords:** Zebrafish, Heart, Regeneration, Model, Myocardial infarction

## Abstract

While humans lack sufficient capacity to undergo cardiac regeneration following injury, zebrafish can fully recover from a range of cardiac insults. Over the past two decades, our understanding of the complexities of both the independent and co-ordinated injury responses by multiple cardiac tissues during zebrafish heart regeneration has increased exponentially. Although cardiomyocyte regeneration forms the cornerstone of the reparative process in the injured zebrafish heart, recent studies have shown that this is dependent on prior neovascularization and lymphangiogenesis, which in turn require epicardial, endocardial, and inflammatory cell signalling within an extracellular milieu that is optimized for regeneration. Indeed, it is the amalgamation of multiple regenerative systems and gene regulatory patterns that drives the much-heralded success of the adult zebrafish response to cardiac injury. Increasing evidence supports the emerging paradigm that developmental transcriptional programmes are re-activated during adult tissue regeneration, including in the heart, and the zebrafish represents an optimal model organism to explore this concept. In this review, we summarize recent advances from the zebrafish cardiovascular research community with novel insight into the mechanisms associated with endogenous cardiovascular repair and regeneration, which may be of benefit to inform future strategies for patients with cardiovascular disease.

## Graphical Abstract

**Graphical Abstract:**
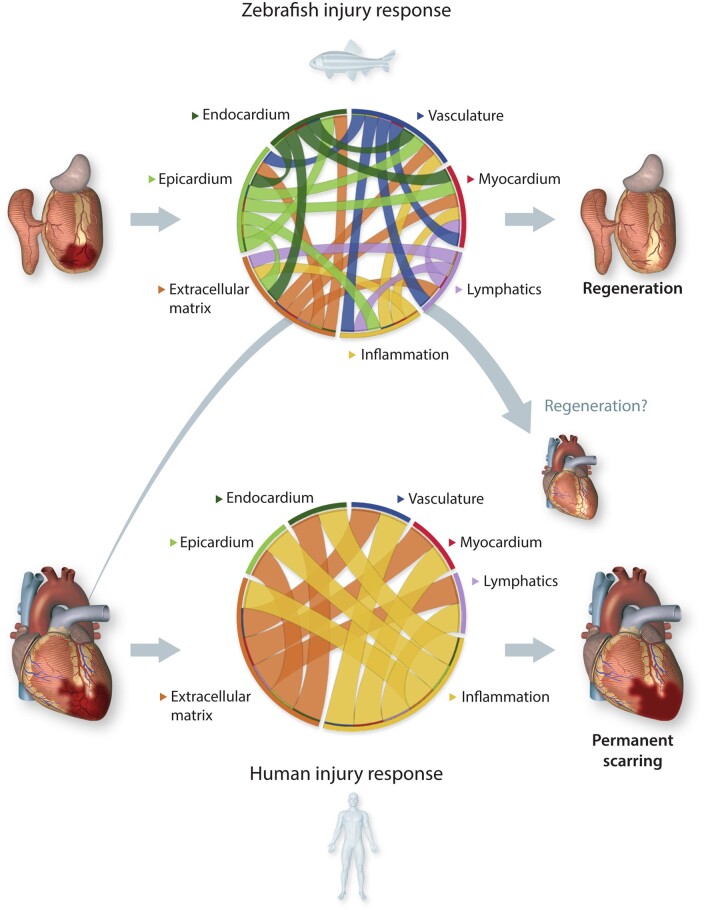
Zebrafish and human injury response linking-diagrams to represent the effects of tissues on one another. Epicardium: light green, Endocardium: dark green, Vasculature: blue, Myocardium: red, Lymphatics: purple, Inflammation: yellow, and Extracellular Matrix: orange. Zebrafish follow a highly integrated cardiac regenerative programme involving all cardiac tissues, illustrated above by the Zebrafish Injury Response linking diagram. This is in direct contrast to humans where ischaemic injury is predominantly followed by inflammation and extracellular matrix deposition, as illustrated in the Human Injury Response linking diagram, leading to permanent scarring. It is interesting to consider whether a full understanding of the efficient regenerative programme of the zebrafish could inform future heart regeneration studies in humans (Krzywinski MI, Schein JE, Birol I, Connors J, Gascoyne R, Horsman D, Jones SJ, Marra MA. Circos: An information aesthetic for comparative genomics. Genome Res 2009; 31:1639-1645).

## 1. Introduction

In humans, myocardial infarction (MI) arising from obstruction of the coronary circulation engenders massive cardiomyocyte loss, leading to pathological remodelling and cardiac dysfunction.^1,2^ Patient care and survival following acute MI have improved substantially^3^ but the lack of targeted approaches for effective repair leads to poor long-term heart function, often resulting in deterioration to heart failure.^1,2^ This is exacerbated by the poor regenerative capabilities of adult mammalian heart, where necrotic and apoptotic tissues are replaced by a permanent non-contractile fibrotic scar. In 2002, Poss *et al.*^4^ first reported the remarkable regenerative capabilities of the zebrafish heart following ventricular amputation. Since then, additional zebrafish heart injury models, such as cryoinjury,^5–7^ genetic ablation,^8,9^ hypoxia,^10^ and laser-induced injury^11^ have been developed and the regenerative responses assessed. Across all zebrafish cardiac injury models, cell death in the injury area is followed by a rapid proliferative response in the endocardium,^12^ epicardium,^13^ myocardium,^14–16^ and vasculature,^17,18^ coupled with a complex temporally controlled inflammatory response.^19^ During the first 3 weeks post-injury, debris is cleared from the injury area and replaced by fibrotic tissue, akin to the mammalian response.^20^ This fibrotic tissue is subsequently degraded and replaced by electrically coupled vascularized myocardium. Though zebrafish are phylogenetically distant from mammals, there are distinct similarities in the developmental pathways^21,22^ and electrophysiology^23^ of their cardiovascular systems and the common evolutionary origin of the heart has been clearly illustrated.^24^ Zebrafish are especially well suited to cardiovascular studies (reviewed by Kithcart and MacRae, 2017^25^) as a result of the high similarity between human and zebrafish cardiac action potentials. Zebrafish have also been used for high-throughput drug discovery and compound screening for preclinical toxicity assessments,^26,27^ resulting in a number of compounds progressing to clinical trials (reviewed by Cully, 2019^28^). Personalized zebrafish ‘avatars’ are additionally gaining traction. Here, patients’ diseases—particularly cancer or rare diseases—are modelled in the zebrafish to provide a platform for developing novel combination treatments, identifying predictive biomarkers, and providing insights into potential treatment resistance mechanisms (reviewed by Costa *et al.*, 2020^29^). While toxicity screens and disease avatars validate the zebrafish as a relevant translatable model, there remains a lack of evidence for the ‘bench to bedside’ pathway for pro-regenerative compounds identified in zebrafish, and plenty more work must be done to fully understand how the zebrafish regenerative programme progresses. With that said, their quick generation time, large clutch sizes, affordability, ease of genetic manipulation, high degree of genetic conservation with humans, and endogenous regenerative capabilities are rapidly making zebrafish the model of choice for many heart regeneration studies.

## 2. Zebrafish cardiac injury models

### 2.1 Ventricular resection

In this model, 20–25% of the ventricle is removed surgically with fine scissors,^4,30^ causing the heart to bleed profusely. The reduced pressure–volume of the trabeculated zebrafish heart is such that blood clotting is sufficient to patch the area of injury. There is then transient collagen deposition, which is eventually resorbed and replaced by healthy contractile cardiomyofibrils. Full recovery of the ventricle is observed by 30–60 days post-injury.^4,30^ Since ventricular resection involves the removal of tissue, there is less requirement for clearance of debris, which may account for the lack of scarring in this injury model (Table 1). Instead, ventricular resection is typically accompanied by an accumulation of apoptotic cells at the border of the injury zone that does not extend further into the wound site.^6^

**Table 1:** Table to show advantages and disadvantages of zebrafish cardiac injury models.

Injury	Schematic	Advantages	Disadvantages	References
Ventricular resection	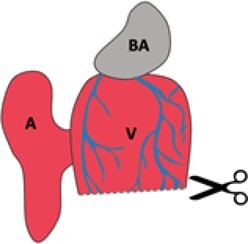	Fast recovery Common in literature Injury to all cell types	No lymphatic regeneration Less similar to human MI Technically challenging Variable injury size Open chest model	Poss, Wilson & Keating, 2002^4^; Raya *et al.*, 2003^30^
Cryoinjury	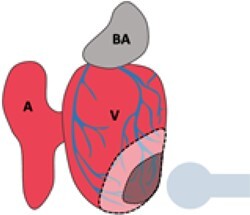	Common in literature Most similar to human MI Injury to all cell types	Long recovery Technically challenging Open chest model	Chablais *et al.*, 2011^5^; González-Rosa *et al.*, 2011^6^; Schnabel *et al.*, 2011^7^
Genetic ablation	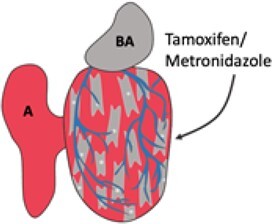	Cell-specific study Non-invasive Fast recovery Technically simple	Limited to single-cell type Less similar to human MI	Curado *et al.*, 2007^8^; Wang *et al.*, 2011^9^
Hypoxia/reoxygenation	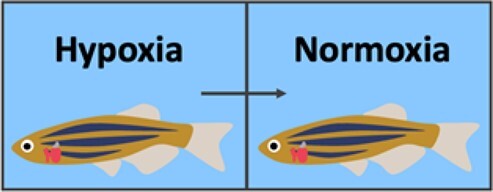	Reperfusion injury Non-invasive Similar to human MI Technically simple	No localized version currently available	Parente *et al.*, 2013^10^
Explant culture	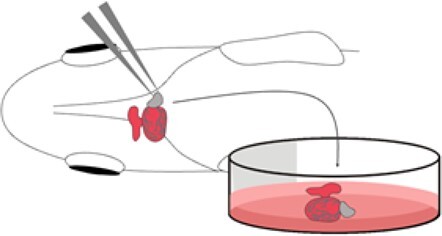	Fewer ethical concerns No requirement for home office approval Live imaging	Loss of circulating factors Declines in heart function Injury response from extraction	Hecker *et al.*, 2008^32^; Pieperhoff *et al.*, 2014^34^; Cao and Poss., 2016^35^; Yip *et al.*, 2020^36^
Laser injury	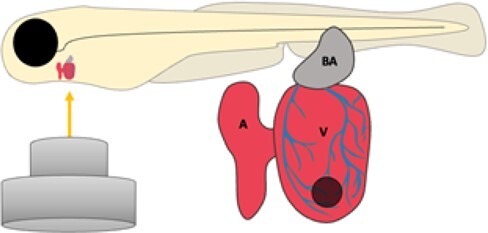	Non-invasive Consistent injury Fewer ethical concerns No requirement for home office approval Live imaging High *N*-numbers	Limited to larval stages Requirement for specialized equipment	Matrone *et al.*, 2013^11^; Kaveh *et al.*, 2020^37^

### 2.2 Cryoinjury

Three seminal papers published in 2011 described the development of a cardiac cryoinjury model in the zebrafish.^5–7^ Cryoinjury has since been gaining popularity in adult zebrafish heart studies as it most closely mimics human MI (Table 1). The pericardial sac is opened and 20–25% of the ventricle is frozen using a pre-cooled cryoprobe. The rapid tissue cooling results in the creation of intracellular ice crystals, which are destructive to the cells. A uniform central region of coagulation necrosis then forms at the probe contact site.^31^ Towards the peripheral border zone, there is a region of apoptotic cell death where the temperature was not sufficiently cold to kill the cells but was cool enough to cause irreparable damage.^31^ Cryoinjury results in widespread fibrosis, necrotic cell death,^7^ and rapid enucleation of cardiomyocytes, whilst leaving the sarcomeric structure intact.^6^ Following cryoinjury, the zebrafish ventricle undergoes cardiac remodelling, taking on a rounded appearance with enlargement of the injured ventricle and concomitant wall thickening.^5,9^ During the first 3 weeks post-cryoinjury, necrotic tissue cell debris is cleared and replaced by transient fibrosis, which is subsequently resorbed and exchanged for functional cardiac tissue.^6^ Importantly, the pathophysiological consequences of cryoinjury are observed in all cardiac cell types, which is in contrast to genetic ablation, discussed below.

### 2.3 Genetic ablation

Cardiomyocytes can be genetically manipulated to express toxins or enzymes that catalyse the production of cytotoxic metabolites. This causes cell-type-specific tissue ablation (Table 1). The first genetic ablation model in larval zebrafish was designed by Curado *et al.*,^8^ in 2007, where cardiomyocytes were genetically altered to express nitroreductase (NTR). NTR—through its catalysation of metronidazole (Mtz)—forms toxins that result in cell death. Wang *et al.*^9^ created a similar injury model in the adult zebrafish whereby cardiomyocytes conditionally expressed the recombinase-responsive diphtheria toxin chain A. The destruction of a high percentage of cardiomyocytes activated robust injury responses by surrounding cell types including a full immune response. Through the control of cardiomyocyte ablation by the addition and removal of Mtz^8^ or tamoxifen^9^ from the fish water, these systems offer highly reproducible temporal control of the injury as well as the ability to induce significant damage non-invasively. Interestingly, the massive loss of cardiomyocytes results in disrupted electric conduction in the ventricle and induces symptoms of heart failure commonly experienced by humans, including fatigue and impaired swimming speed and endurance.^9^ However, in this model only a single-cell type is affected and there is a likelihood of a non-specific ‘bystander effect’ on neighbouring cell types. Unlike the other injury models explored in this review, the full cohort of destroyed cardiomyocytes in the Wang *et al.*^8,9^ adult genetic ablation model is fully regenerated after just 30 days. This suggests that the maintenance of other cell types improves regeneration times by weeks, despite a greater proportion of the muscle being injured (>60% in genetic ablation compared to 20–25% in ventricular resection or cryoinjury). It is interesting, however, that the zebrafish myocardium can be damaged to the extent that the fish enters a state resembling heart failure but can still be rescued by its intrinsic regenerative capacity.^9^

### 2.4 Hypoxia/reoxygenation

Of course, the model to most closely resemble human MI would be a localized induction of hypoxia. Unfortunately, no such model currently exists in the zebrafish. However, in 2013, Parente *et al.*^10^ established a global hypoxia/reoxygenation model (Table 1). While effective in reproducing hypoxia response mechanisms, this model stimulates hypoxia-induced injury that is not limited to the heart. There is evidence of inflammation and apoptosis in other organs and a global activation of hypoxic response pathways. After induction of this injury model, the zebrafish displays a transient reduction in cardiac function but the injuries to the heart are not severe enough to be visible histologically.^10^ It is interesting to note that a degree of hypoxia is actually required to stimulate the regenerative response. Jopling *et al.*^15^ showed that exposure to hyperoxia dramatically impeded zebrafish heart regeneration, while hypoxia improved cardiomyocyte proliferation. Although the global hypoxia model certainly induces the injury/regeneration response, the quest to establish a localized hypoxic model in the zebrafish heart continues.

### 2.5 Explant culture

In 2008, Hecker *et al.*^32^ performed a functional and histological evaluation of explanted adult zebrafish hearts. They found that hearts that were sectioned immediately and 1.5 h after isolation were histologically indistinguishable,^32^ thus concluding that the hearts remained viable outside the body. Over this time, the hearts additionally maintained spontaneous contractile properties. This offered evidence that the isolated zebrafish heart could provide a powerful, yet simple, tool for assessment of cardiovascular function. In 2010, this system was adapted to evaluate the role of Pdgf in epicardial regeneration in the adult zebrafish heart.^33^ Here, injured and control ventricles were extracted and cultured on a fibrin gel. Recombinant Pdgfb and its inhibitors were added directly to the culture medium to create an *ex vivo* over-expression and rescue model, showing a role for Pdgf in mediating the stress response after injury.^33^ Two independent studies have examined the responses of the explanted heart to isoproterenol^32,34^—also known as isoprenaline—which is an epinephrine analogue commonly used in humans for the treatment of bradycardia. In both studies the heart rate increased, as expected, in response to the drug, highlighting the relevance and extended potential of an explant system that could aid identification of small molecules to benefit the regenerative response.^4^ To this end, in 2016, Cao and Poss^35^ published a protocol to culture live zebrafish hearts—both injured and healthy—for up to 30 days. Like Kim *et al.*, they used this system to investigate epicardial regeneration and to identify therapeutic targets for heart disease. This culture system introduces an array of novel research opportunities that were previously inaccessible due to the opacity of the adult fish. Indeed, while zebrafish larvae are celebrated for their transparency, in the adult zebrafish repair is primarily assessed using histology, precluding repeat investigations of the same animal. Tissue explants could therefore provide a more physiologically relevant alternative to traditional cell culture experiments (Table 1). Potential drawbacks of *ex vivo* assays, however, include a loss of circulating factors and inflammatory responses that will undoubtedly impact the regenerative process. Further, explanted hearts experience declines in function within 72 h.^34,36^ This can be somewhat alleviated by the introduction of agitation in culture,^35^ but this is not compatible with continuous live imaging techniques. To address this, Yip *et al.*, 2020,^36^ designed a microfluidics device that significantly reduced structural and functional declines observed in other culture methods and permitted continuous live imaging for over 4 days. As we continue to observe improvements in this model, it is important to consider the role it may play in reducing the number of *in vivo* studies that may cause lasting harm or suffering to the fish.

### 2.6 Laser injury

Matrone *et al.*, 2013,^11^ developed a laser-induced injury method that is less technically challenging than the other more common and more intricate resection and cryoinjury models with the added bonus of being completely sterile, highly accurate and requiring a smaller timeframe for full functional recovery (Table 1). They showed, both functionally and histologically, that this injury method produces an injury of sufficient severity to induce a full regenerative response.^11^ This injury model has since been effectively utilized for *in vivo* mapping of immune cell recruitment to the heart after injury *via* live imaging techniques.^37^ Unfortunately, this model relies on the transparency of the larval zebrafish and no adult adaptations have thus far been reported. It is, however, interesting to consider the possibility of combining the continuously improving explant culture with this promising injury model to create an *ex vivo* adult regenerative response assay with simultaneous live-imaging opportunities.

## 3. Cardiomyocyte regeneration in the zebrafish heart

One of the cornerstones of heart regeneration is the repopulation of functional cardiomyocytes into the injury area. This review explores the mechanisms that drive this in the zebrafish and addresses the interconnectivity of multiple systems within the heart that are required for successful cardiac regeneration. The genetic signalling programme responsible for driving zebrafish heart regeneration at least in part recapitulates heart development.^14,38^ Given conservation of many of these mechanisms, this supports comparative studies between species to better understand potential pathways for organ regeneration in humans. Therefore, understanding the origin of the cells that contribute to the regenerating myocardium as well as the transcriptional landscape of regenerating zebrafish hearts may aid in identifying areas where mammalian injury responses fall short. In 2006, the first study aimed at addressing the source of regenerative cardiomyocytes in zebrafish revealed that these cells arise from an undifferentiated progenitor cell population.^14^ Later, in 2010, Cre/Lox genetic fate-mapping suggested that pre-existing lineage-committed cardiomyocytes were the predominant contributors to myocardial regeneration after ventricular resection.^15,39^ The cardiomyocyte-origin of the regenerated myocardium has since been confirmed in multiple zebrafish injury models.^7,11,40^ Likewise, neonatal mouse cardiac regeneration is achieved through the re-entry of cardiomyocytes into the cell cycle rather than through differentiation of cardiac progenitors.^41^ Studies using ^14^C isotope dating have revealed that human cardiomyocytes continue to undergo proliferation at a very low rate (<1% per year) into adulthood.^42,43^ Though this cell turnover is not sufficient to support regeneration, the potential for amplifying the proliferative process in the context of regeneration is intriguing. In the zebrafish, proliferating cardiomyocytes undergo a degree of dedifferentiation that includes disassembly of sarcomeric structures, reduced expression of mature sarcomeric proteins and concomitant up-regulation of developmental myosins^44,45^ as well as changes to cell adhesion molecules and re-entry into the cell cycle.^15,39^ In line with reports that border zone cardiomyocytes dedifferentiate in response to injury,^15,45^ Koth *et al.*, 2020,^46^ showed an up-regulation of developmental myosin heavy chain 7 (*myh7*) in the regenerating heart using single-cell RNA-sequencing (scRNAseq). Similarly, a portion of cardiomyocytes from the subepicardial region reactivate cardiomyocyte developmental marker *gata4* regulatory sequences. These cells preferentially contribute to the regenerating myocardium, showing reactivation of developmental gene programs in mature cardiomyocytes.^39^ This is further supported by the abrogation of injury-induced cardiomyocyte proliferation in response to the expression of a dominant-negative form of developmental gata4.^47^ For a comprehensive overview of the signalling pathways activated during zebrafish cardiac regeneration, the authors recommend González-Rosa *et al.*, 2017^48^ and Pronobis and Poss, 2020.^49^

An essential factor lending itself to the reaction of zebrafish cardiomyocytes to injury is their sustained responsiveness to mitogenic signals into adulthood.^16,44^ Choi *et al.*^50^ performed a chemical screen on zebrafish larvae to identify small molecules that affected cardiomyocyte proliferation during cardiac development. They identified compounds acting via hedgehog (*hh*), insulin-like growth factor (*igf*) and transforming growth factor β (*tgfβ*) signalling pathways that were able to influence the rate of developmental cardiomyocyte proliferation. They then examined the effects of those compounds on cardiomyocyte proliferation in adults following ventricular resection and found that they contributed similarly to myocardial expansion.^50^ Further, it was shown that these small molecules could be pharmacologically manipulated to enhance cardiomyocyte proliferation during adult cardiac regeneration.^50^ This supported previous reports that *igf* signal transduction is required for *gata4*-expressing cardiomyocyte proliferation and contribution to regenerating myocardium.^51,52^ In addition to *igf*, signalling by secreted growth factors, such as fibroblast growth factors,^14,16,53^ platelet-derived growth factors,^33,52,54^ and neuregulins (Nrgs)^39,55,56^ have also been extensively shown to stimulate cardiomyocyte proliferation (reviewed by Pronobis and Poss, 2020^49^). These growth factors function via the Ras/MAPK pathway, which is tightly regulated by the feedback attenuator Dusp6. Missinato *et al.*, 2018,^54^ showed that suppression of *dusp6* enhanced cardiac regeneration following ventricular resection. Inactivation of *dusp6* by small molecules or gene inactivation increased cardiomyocyte proliferation as well as neovasculogenesis and reduced fibrosis. As with *dusp6*, Koth *et al.*^46^ recently recognized *runx1* as a potential proliferation dampener. They showed that *runx1*^−^^/^^−^ mutants had higher cardiomyocyte proliferation and increased cardiomyocyte survival in response to injury than wild-type fish.^46^ This begs the question as to why an efficiently regenerating species like the zebrafish would express genes like *dusp6* and *runx1* that may hamper the regenerative process. The answer may be linked to the increased proliferation and survival of *dusp6*^*−*^^*/*^^*−*^ and *runx1*^*−*^^*/*^^*−*^ cardiomyocytes—phenotypes that are often associated with cancer. It is well known that humans do not suffer from cancers of the heart, likely owing to the significantly reduced proliferative capacity of human cardiomyocytes. Likewise, zebrafish do not develop cardiac cancers despite maintaining myocardial proliferative potential and this may be the result of the expression of proliferative inhibitors, such as *dusp6* and *runx1*. Absence of *runx1* results in an up-regulation of the Annexin2a receptor, a phenotype strongly linked to cancerous proliferation.^57^ Both *runx1* and *dusp6* may therefore function as gatekeepers to maintain control over the proliferative injury response by mitigating an unrestrained multiplication of cells. The (in)activation of Notch receptors located on the endocardium and epicardium following ventricular resection significantly impacted cardiomyocyte proliferation. This shows that cardiomyocytes are highly sensitive to fluctuations in signalling levels, and that the transcriptomic profile following injury must be tightly controlled.^58^ Notch has also been shown to mediate the inflammatory response and the successful regeneration of the endocardium.^12^ Dynamic induction of the *jak1/stat3* pathway in cardiomyocytes is accompanied by cytokine production and a prolific immune response,^47^ once again highlighting the importance of cross-system communication to drive a full and cohesive regenerative programme. In addition, spatial RNA analysis (tomo-seq) of the cryoinjured adult zebrafish heart permitted unbiased genome-wide mapping of region-specific gene expression, highlighting the reactivation of developmental pathways post-injury, and a requirement for bone morphogenetic protein signalling for cardiomyocyte proliferation. A deeper understanding of the transcriptional regulation of regeneration will be vital for future regenerative medicine strategies to aid repair without abetting a malignant proliferative response.

Importantly, the transcriptional landscape is not governed by signalling pathways alone; many of the morphological and transcriptional changes observed are regulated by epigenetic alterations. Indeed, methylation by chromatin-remodelling factor Brg1 is a requirement for successful adult zebrafish heart regeneration.^59^ Transgenic over-expression of a dominant-negative form of Brg1 abrogated cardiomyocyte proliferation, which led to retention of the fibrotic scar. Mechanistically, Brg1 acts *via* up-regulation following injury to interact with methyltransferase Dnmt3a. This leads to down-regulation of cell cycle regulator *cdkn1c* by methylation of the *cdkn1c* promoter.^59^ Similarly, sarcomere and cytoskeletal gene expression in proliferative cardiomyocytes during regeneration has been linked to methylation of H3K27.^60^ Inducing a mutation in histone 3—thereby preventing methylation—results in failed cardiac regeneration in adult zebrafish.^60^ As with Brg1, H3K27me3-mediated gene silencing is essential for adult zebrafish heart regeneration. Thorough exploration into epigenetic chromatin remodelling *via* DNA methylation and histone modifications will augment our understanding of the transcriptional changes at play during regeneration.

Post-transcriptional regulation by way of micro-RNAs (miRNAs) should also not be discounted. Eulalio *et al.,* 2012,^61^ performed a functional screen that identified 40 miRNAs that enhanced neonatal mouse and rat cardiomyocyte proliferation, two of which—miR199 and miR590—were later shown to improve cardiac regeneration in adult mice. The same group later showed that when miR-199 (which is highly conserved in zebrafish^62^) stimulated cardiac repair when exogenously administered to infarcted pig hearts.^63^ This indicates that stimulation of cardiomyocyte proliferation is attainable in large mammals and that miRNAs could be part of the solution. However, persistent expression of miR199 resulted in sudden mortality of most of the treated pigs,^63^ underscoring the importance of gaining a fuller understanding of the cardiac regenerative programme in order to deliver a controlled targeted repair strategy. In 2016, Crippa *et al.*^64^ performed a comparative gene and miRNA profiling of the cardiac transcriptome in mice and zebrafish. They identified 45 miRNA-dependent networks that were evolutionarily conserved but differentially regulated in the mammalian and teleost models. In particular, they found miR-26a—the most abundant miRNA in the zebrafish heart—to be down-regulated following cardiac insult in zebrafish while its levels remained unchanged in the mouse after coronary ligation. miR-26a negatively regulates a number of cell cycle activators and its inhibition was therefore shown to stimulate cardiomyocyte proliferation.^64^ Microarray analyses by Yin *et al.*^65^ identified a similar pattern in a number of differentially expressed miRNAs during adult zebrafish regeneration. One of these—miR-133—targets cell cycle factors *mps1, cdc37*, and *pa2g4* as well as cell junction components *cx43* and *cldn5*, giving an indication to its mechanism of action in activating the proliferative response when down-regulated. Similarly, after ventricular resection miR-99/100 and let-7a/c are down-regulated, resulting in up-regulation of predicted targeted genes, which are highly evolutionarily conserved in zebrafish, mice, and humans.^66^ During development, miR-99/100 and let-7a/c pathway activity is similar between zebrafish and mammals, but after MI, expression levels remain constant in mice but are down-regulated in zebrafish. Interestingly, artificial inhibition of miR-99/100 and let-7a/c in mice after injury—to match zebrafish expression patterns—promotes cardiomyocyte dedifferentiation and proliferation, improving cardiac function and reducing scarring.^66^ miR-101a expression was also associated with the zebrafish cardiac injury response mechanism.^67^ Within the first 3 days after ventricular resection miR-101a levels were dramatically reduced, corresponding with the onset of cardiomyocyte proliferation, but highly up-regulated by 7–14 days, likely to be associated with removal of fibrosis since prolonged suppression of miR-101a was accompanied by cardiomyocyte proliferation but a failure to clear the scar tissue.^67^ It is interesting that in most of these studies, changes in miRNA expression triggered by injury in the zebrafish are not mirrored in mammals, which could point to the contribution of miRNA to the comparatively high regenerative capacity of the zebrafish heart. Further, the easy administration of synthetic anti-miRNAs *in vivo*^68^ may suggest that miRNAs could provide a new avenue to explore in the pursuit of mammalian cardiac regenerative medicine strategies.

A number of other factors merit consideration in zebrafish cardiac regenerative ability but are beyond the scope of this review. Regenerative potential of cardiomyocytes can be influenced by their oxidative status or by stress^44^ as well as by genes involved in mitochondrial regulation.^69^ Environmental and systemic factors, such as overcrowding also impact regeneration^44^ as well as hemodynamic shear stress (reviewed by Li *et al.*, 2019^56^), which affects cardiomyocyte and endothelial cell activity post-injury. Another interesting contribution to cardiomyocyte proliferation is that of nerves. Mahmoud *et al.*, 2015,^70^ showed that the inhibition of cardiac innervation resulted in impaired cardiomyocyte proliferation and regeneration. It will be interesting in the coming years to gain a fuller picture of how these systems contribute to the ultimate goal of full cardiac regeneration.

## 4. Vascular regeneration

### 4.1 Coronary neovascularization

Neovascularization has been shown to be essential for myocardial regeneration in zebrafish^6,17,18,71^ and it has been postulated that regenerative neovascularization recapitulates larval coronary vessel development. This is supported by a 2010 study, where adult zebrafish hearts were treated with a Pdgfr inhibitor to show that *pdgf* signalling plays a crucial role in reactivating developmental angiogenic transcription pathways during regeneration.^33^ Interestingly, this *pdgf*-dependent regenerative response appears to be conserved across species as epicardium-derived *Pdgfrb* knockout mice display defective coronary artery formation.^72^ Marín-Juez *et al.*, 2016,^17^ used lineage tracing to reveal that the primary source of regenerated cardiac vasculature in adult zebrafish is pre-existing coronary vessels. They found that cryoinjury in adult hearts expressing the endothelial-specific transgene *fli1a: GFP* resulted in first vessel sprouting at 15 h post-injury and that these sprouts formed as GFP^+^ extensions of vessels saved from the injury. To investigate previous reports that epicardial cells were contributing to the new vasculature,^14^ they then repeated the experiment using fish harbouring the *tcf21: DsRed2* transgene, which labels epicardial and epicardial-derived cells (EPDC), and found no DsRed2^+^ cells in the newly regenerated vasculature at any timepoint. Building on work by Harrison *et al.*^71^ showing that during development migratory vessels express *cxcr4a* whose expression is later restricted to arteries, Marín-Juez *et al.*^17^ observed that revascularization of the damaged area follows a signalling pattern that mimics the developmental programme. Zebrafish have two Cxcl12-encoding genes—*cxcl12a* and *cxcl12b—*and two genes encoding receptors—*cxcr4a* and *cxcr4b.*^73^ The migration of *cxcr4b*-expressing endothelial cells along a Cxcl12 gradient is an established pro-angiogenic developmental pathway.^73^ Harrison *et al.*, 2015,^71^ showed that *cxcr4a* mutant zebrafish hearts no longer displayed regenerative potential into adulthood in response to ventricular resection. Instead, mutant fish formed scar tissue that was not resolved by 120 days post-amputation.^71^ Similarly, in 2019, Marín-Juez *et al.*^18^ used loss- and gain-of-function experiments to show that superficial revascularization in the cryoinjured adult zebrafish heart is regulated by epicardial *cxcl12/cxcr4* signalling. They showed that *cxcl12b* is expressed only in the activated epicardium lining the injured area following cryoinjury and that genetic and chemical inhibition of Cxcr4a resulted in blockage of superficial coronary revascularization.^18^ The *cxcl12/cxcr4* pathway has also been implicated in cardiomyocyte regeneration,^74^ emphasizing the inter-functionality of regenerative signals. As with *pdgf, cxc* signalling appears to be evolutionarily conserved as mouse *Cxcr4* and *Cxcl12* knockouts also show developmental vascular defects.^75^ It is interesting that the expression of *cxcl12b* by the epicardium appears to be induced by hypoxia *via* Hif1a.^18^ Using hypoxia-related mRNA quantification and a hypoxyprobe, it was shown that ventricular epicardial cells are extremely hypoxic at 7 days post-injury and that there were significantly reduced *cxcl12b* mRNA levels in Hif1a deficient hearts compared to wild type controls.^18^ Similarly, in *hif1a*^−^^/^^−^ ventricles there was a significant reduction in proliferating cardiac endothelial cell numbers. In contrast, stabilizing Hif1a resulted in increased endothelial cell proliferation and improved cardiac vessel sprouting, indicating that through hypoxia—an environment that is innate to ischaemic injuries—*cxcl12b* is activated in the epicardium to drive vascular regeneration. Indeed, *hif1a* is not the only hypoxia-related pathway that has been implicated in adult zebrafish cardiovascular regeneration. Earlier this year, it was described how nitric oxide—the reduced form of nitrite, which occurs under hypoxic conditions—improved angiogenesis, immune cell recruitment, and cardiomyocyte proliferation following both cryoinjury and ventricular resection.^76^

In addition to epicardial *cxc*-dependent superficial sprouting, Marín-Juez *et al.*^17,18,38^ also showed that deeper intra-ventricular coronary sprouting after cryoinjury was regulated by *vegfaa*, which is expressed in the activated endocardium. As early as 15 h post-injury, *vegfaa* expression was observed in the endocardium, coinciding with the initiation of revascularization, and this expression persisted for the duration of active cardiac endothelial cell proliferation following injury.^18^ Knockdown of *vegfaa* dramatically impaired intra-ventricular sprouting, while *flt1*^*−*^^*/*^^*−*^ fish, which exhibit excessive *vegfaa* signalling, displayed a greater number and length of intra-coronary vessels.^18^

### 4.2 Lymphatic regeneration

**Figure 1:**
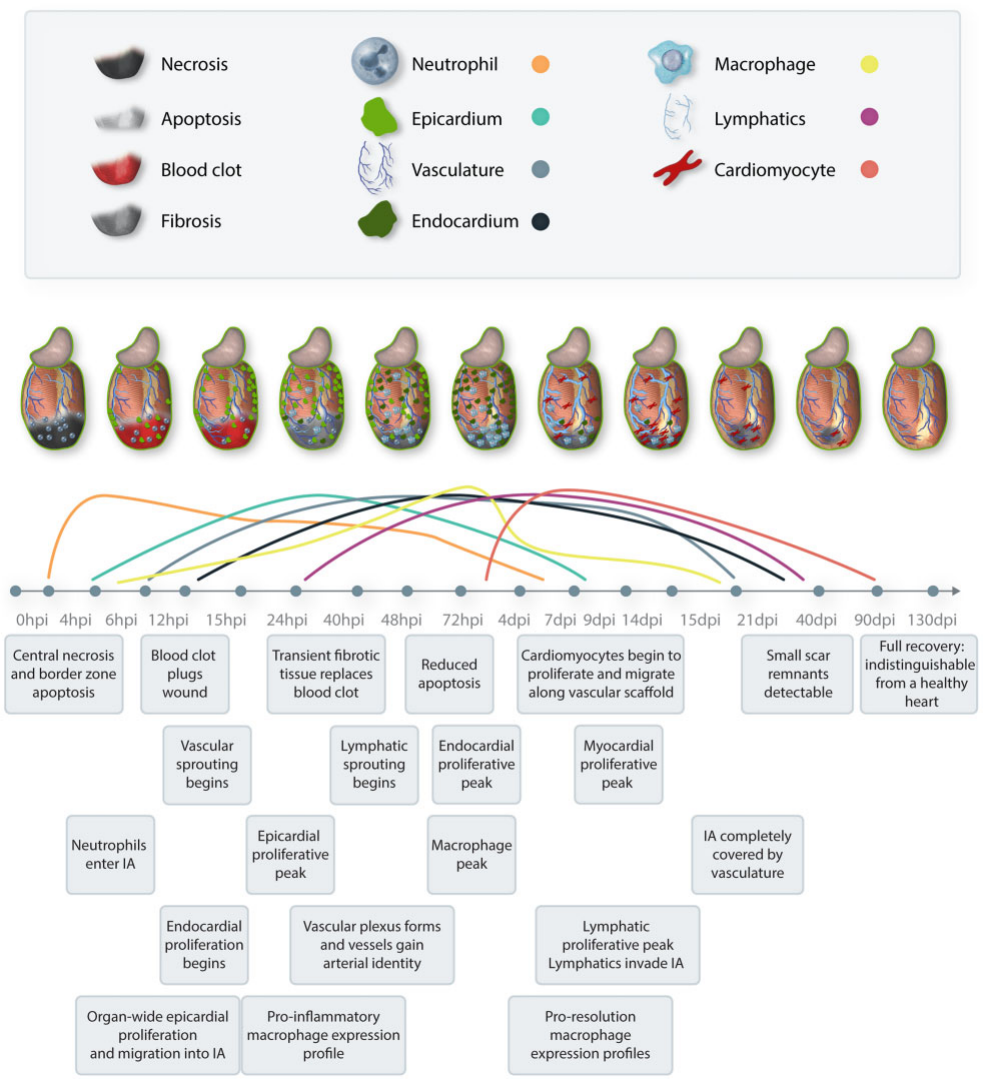
Overview of the cardiac regenerative timeline of the adult zebrafish heart. Top panel shows graphical representation of regenerating structures in the injury area. The second panel shows relative waves of regeneration to compare activation timings across structures. The bottom panel shows timing of these events in hours post-injury and days post-injury, citing key references, and highlighting interaction of systems at each time point.

Regeneration of the lymphatic vasculature is another important feature of zebrafish heart regeneration, which also tends to follow development-associated patterns.^77–80^ Failure of the lymphatic system to regenerate results in significant scarring and immune cell retention in the zebrafish heart after cryoinjury,^71,77^ highlighting how the cardiac lymphatic system influences myocardial regenerative capabilities by facilitating clearance of scar tissue and immune cell debris. In 2019, Harrison *et al.*^77^ performed intramyocardial injection of microspheres and quantum dots to visualize lymphatic vessel development; they found that cardiac lymphatic vessels developed using coronary arteries as a scaffold. This process was mimicked in a regeneration setting^77^ and *vegfc/vegfr3*^71^ and *cxc/cxcr*^71,77^ mutant fish, which are unable to form cardiac vasculature, also failed to develop functional lymphatic systems. Vieira *et al.*, 2018,^81^ were the first to show that resolution of inflammation following MI in mice is stimulated by the cardiac lymphatic system *via Vegfc.* This led to follow-up studies in the zebrafish that have confirmed that in a regenerative setting cardiac lymphangiogenesis is stimulated by Vegfc to improve debris clearance from the wound site and when Vegfc ligands were inactivated in the lymphatic vasculature prior to cryoinjury, there was an overwhelming increase in *mpx^+^* neutrophils left in the wound site as late as 2 weeks post-injury.^77^ Interestingly, lymphatic vessels efficiently sprout into the injury area following cryoinjury in adult zebrafish hearts but less so in response to ventricular resection.^77^ This is possibly due to the reduced inflammatory response and lack of scarring that tends to accompany ventricular resection, negating the necessity for lymphatic regeneration. Given the requirement for established vasculature before lymphangiogenesis can begin,^77^ there is a resulting lag in the timing of lymphatic regeneration compared to the coronaries (*Figure 1*).

## 5. Epicardium and endocardium

The epicardium and the endocardium are two of the first structures to undergo regeneration in response to injury in zebrafish (*Figure 1*), preceding the responses of the myocardium and the coronary vasculature.^12,13,46,48,82^ They form active scaffolds to provide mechanical and paracrine support to guide regeneration of the other cardiac tissues.^13,18^ Immediately after injury, both the epi- and endocardia are activated in an organ-wide response.

### 5.1 Epicardium

Given the strong signalling roles fulfilled by the epicardium, it is no surprise that epicardial ablation results in impeded cardiomyocyte proliferation and delayed cardiac repair.^83^ The epicardium undergoes morphological changes in immediate response to injury,^13^ which are accompanied by an induction of developmental signalling markers that are not expressed in uninjured adult zebrafish.^6,7,14,50^ This includes retinoic acid synthesizing enzyme, *raldh2*, and developmental transcription factors *tbx18* and *wt1.*^6,7,14^ The activation of these markers is initially observed organ-wide, but by 3 days post-injury, expression becomes restricted to the injury area.^7,13^ There is also an epicardial paracrine release of developmental signals^18,55,74,84^ to contribute to cardiomyocyte and vascular regeneration and up-regulation of known stimulators of cardiomyocyte proliferation.^47^

Epithelial to mesenchymal transition (EMT), wherein the epicardium forms EPDCs, is another essential component of zebrafish heart regeneration. The epicardial cells lose their adhesions and accumulate in the wound where they undergo EMT to create a thickened ‘epicardial cap’ over the injury site.^6,33,85,86^ EMT markers, *snail* and *twist*, are present in cryoinjured but not sham-operated hearts,^33^ indicating that EMT is exclusive to an injury setting in the adult heart. Following EMT, EPDCs migrate into the wound area and differentiate into perivascular cells and myofibroblasts.^15,38^ Kikuchi *et al.*^87^ showed through Cre/Lox genetic fate-mapping that after injury, EPDCs also contribute to the smooth muscle of the bulbus arteriosus. In addition to providing a source of paracrine signalling and acting as a cell reserve, the epicardium is a mediator for inflammation^14,83,86–88^ and strongly expresses extra cellular matrix proteins, such as fibronectin, periostin, collagen I, and collagen XII to modulate the extracellular environment to be optimized for regeneration.^13,55^

### 5.2 Endocardium

Less is known about the role of the endocardium, though it is clear that it contributes to the structure of the ECM in the regenerating heart.^12,82,89^ Shortly after induction of epicardial regeneration, the endocardium begins to proliferate rapidly to form a regenerating sheet on the inside of the wound.^12^ The endocardial cells also begin their injury response by undergoing major morphological changes. The cells become rounded and partially detach from the surrounding myocardium whilst extending actin-rich filopodia-like protrusions to acquire a motile phenotype at 24 h after injury.^12,38^ These cells are then reported to spread across the injury area and reorganize into a cohesive sheet to form the new regenerated endocardium after 9 days with an endocardial proliferative peak occurring at 3 days post-injury, just prior to the 7 days post-injury proliferative peak for cardiomyocytes^12^ (*Figure 1*). Like the epicardium, the endocardium plays an important role in controlling the extracellular matrix (ECM). scRNAseq data from Koth *et al.*^46^ in 2020 indicated that much of the collagen deposition in the wound was found to be adjacent to *myh11*-expressing endocardial cells and thrombocytes. This observation is consistent with previous reports that the endocardium proximal to the wound edge up-regulates collagens to contribute to the deposition of scar tissue.^12,82^ This suggests that while endocardial cells retain their original cellular identity during regeneration, they may function analogously to myofibroblasts in the way that they contribute to scar tissue deposition. The dual identity of these collagen-expressing endocardial cells may suggest that the injury-induced fibrosis observed in the zebrafish is driven by a more transient and less differentiated cell population than myofibroblasts.^46^ It has been postulated that because of this, the collagen deposits are less stable and easier to degrade, which contributes to the transient nature of the zebrafish scar tissue.^46^ This, however, is largely speculative and requires further investigation.

## 6. Inflammatory response

The importance of temporally controlled tissue inflammation and immune cell recruitment as an immediate response to injury is well documented.^19,90–92^ It is known that a dampened immune response after cryoinjury strongly impairs cardiomyocyte mitotic activity^91,93^ and diminishes angiogenesis.^90,93^ Neutrophils and pro-inflammatory cytokines are observed in the wound area as early as 3 h post-injury.^51^ However, while neutrophils play a crucial role in the tissue repair process as first responders, they also generate reactive oxygen species and secrete proteases before undergoing apoptosis.^94^ For this reason, spatial confinement and timely clearance are critical to prevent further tissue damage. Circulating macrophages infiltrate as the second wave responders in the immune response, peaking at 7 days after injury.^93^ Canonically, these cells aid in optimizing the extracellular niche by releasing pro-inflammatory cytokines, mediating ECM turnover, activating cardiac fibroblasts, and clearing necrotic debris *via* release of proteolytic enzymes and phagocytosis. They are also responsible for the clearance of neutrophils from the injury location.^93^ Bevan *et al.*, 2019,^19^ showed that *tnfa*^+/+^ macrophage clearance of neutrophil debris also aided the deposition of the collagenous tissue during the early phases after cryoinjury and that *tnfa*^−^^/^^−^ macrophages facilitate scar removal later in the inflammatory process. Linking with the role of the efficiently regenerating lymphatic vasculature, elimination of immune cell debris is a key step in the regenerative process to maintain an extracellular environment that is conducive to healing^77,81,93^ and indeed, lymphatic regeneration is at its highest just after the macrophage peak around 7 days after injury (*Figure 1*). A key study by Simões *et al.*,^92^ revealed that macrophages directly contribute collagen to the ECM following injury—a phenomenon that they found to be conserved in both zebrafish and mice. The importance of the macrophage response in regulating the ECM has also been highlighted in a number of macrophage ablation studies. Early macrophage ablation by clodronate liposomes caused reduced collagen deposition at the injury site, while late macrophage ablation resulted in failure of scar resolution.^19,91,93^ There are a number of molecular mechanisms that may mediate this precisely phased inflammatory response including matrix metalloproteases (MMPs),^52,95^*cxcl8* and *ccl2* signalling^95^ and Toll-like receptor signalling.^93^ Given that well-timed inflammation aids rather than inhibits regeneration,^7,9^ remarkably little is currently known about the regulation of inflammatory signals in the injured zebrafish heart.

## 7. ECM remodelling

The ECM is a network of proteins responsible for the structural integrity of the myocardial tissue that allows for electrical transmission between the cardiomyocytes. After injury, fibrosis develops at sites of cardiomyocyte necrosis in order to preserve the structural integrity of the myocardium. In humans, this is a permanent fibrotic response that results in adverse functional consequences, which can progress to heart failure.^96^ Persistence of a fibrotic scar is therefore often cited as the key difference between the non-regenerative human response and the efficient recovery of the zebrafish heart. Immediately following cardiac injury in the zebrafish, a fibrin clot forms over the injury site, which is rapidly followed by the accumulation of myofibroblasts. These myofibroblasts are responsible for modulating the ECM by expressing proteins, such as collagens, fibronectin, vimentin, and tenascins and by controlling the electrical coupling and contractility of adjacent cardiomyocytes.^5,55,97,98^ Importantly, deposition of ECM components also function to alter the tension of the tissue, which can induce signalling *via* mechanosensitive pathways.^56,97^ Wang *et al.*, 2013,^55^ applied a proteomics approach to show that fibronectin—a major ECM protein—is deposited by the epicardium after cardiac injury in the adult zebrafish. They showed that two fibronectin paralogues are expressed in epicardial cells following an injury event, while the IntegrinB3 receptor is expressed in surrounding cardiomyocytes. Through the integrin receptors, extracellular signals from the fibronectin are transduced, though they do not appear to make a direct contribution to cardiomyocyte proliferation during the repair process. Instead, it appears that fibronectin is required for cardiomyocyte mobilization and integration into the injury zone.^55^ Similarly, integrins on the basal surface on endothelial cells connect them to ECM components and act as mechanosensors to influence the development of new blood vessels.^56^ The observation that macrophages,^92^ epicardial,^55^ and endocardial cells^46^ also contribute collagen to this tissue challenges the dogma that myofibroblasts are the sole source of scar tissue post-cardiac injury and emphasizes the requirement for multi-tissue interactions during regeneration. Due to the persistence of scarring in adult mammals, the fibrotic response is often considered to be inhibitory to regeneration, but this has unequivocally been shown not to be the case.^6,40,82,99^ In fact, it is probable that fibrosis has been evolutionarily selected. Indeed, limiting the fibrotic response during the early phases after injury results in failed regeneration.^40,82^

Molecularly, the expression of key factors to control the intricate scarring process remains largely unexplored. Chablais *et al.*^40^ showed that part of the balance between scar resolution and persistence is controlled by *smad3*-dependent *tgfβ/activin* signalling. The expression of type I receptor *alk5β* (*tgfr1β*) is seen in both the cells of the fibrotic scar and the neighbouring cardiomyocytes. Ligands for these receptors are locally expressed during cryoinjury thereby activating the *tgfβ* signalling pathway in both the injury zone and the cardiomyocytes surrounding the trauma. Blocking this pathway through chemical receptor inhibition resulted in failure of the heart to regenerate at all. Using this reversible inhibitory approach, the researchers were able to identify three critical stages of zebrafish heart regeneration, which include (i) the deposition of a transient collagen-rich scar, (ii) Tenascin C-associated tissue remodelling at the infarct border, and (iii) cardiomyocyte proliferation.^40^ It is interesting to note that of the three required processes for successful regeneration, two of them pertain to ECM structural remodelling.

In addition to *smad/tgf* signalling, it is also clear that many of the components of the fibrotic ECM are broken down by MMPs. The action of MMPs in the injury area helps to control not only ECM turnover, but also contributes to inflammatory signalling, which in itself modulates the extracellular environment by clearing necrotic tissue.^100^ Collagenolytic activity in the regenerating adult zebrafish heart is primarily regulated by MMP2 and MMP14a,^101^ whose activity decreases as the myocardium regenerates. Expression of the genes coding for MMP2, MMP14a, and MMP14b begin at 7 days post-injury and last until 14 days post-injury, suggesting that ECM remodelling occurs in the middle stages of zebrafish heart regeneration. In a comparison between heart and fin regeneration, tissue remodelling genes made up the bulk of the gene expression categories that matched in the two zebrafish regeneration models.^52^ Comprehensive mining of DNA microarrays and Gene Ontology term enrichment analyses for regenerating zebrafish hearts by Mercer *et al.*^98^ revealed that distinct ECM components and ECM-modifying proteases are among the most significantly enriched genes in response to local injury. In contrast, data analyses for mammalian cardiac injury models indicated that inflammation and metabolic processes are the most significantly activated gene groups.^98^ It is worthy of note that the zebrafish and mammalian responses to injury do not differ significantly in the early stages, but where mammals present with prolonged inflammation and retention of scarring, these phases are transient in the zebrafish.

Elimination of profibrotic cells has been postulated to progress *via* apoptosis^102^ but TUNEL staining of *postnb^+^* fibroblastic cells reveals that there is remarkably little cell death following injury. Sánchez-Iranzo *et al.*, 2018,^82^ showed using lineage tracing and RNA-sequencing that during fibrosis regression, the fibroblasts are not eliminated but instead persist in the wound site but in a deactivated state. While still present, the *postnb^+^* cells drastically reduce expression of ECM-associated genes although *col7a1l* and *col8a2* remain up-regulated from 7 to 60 days post-injury compared to uninjured control hearts, indicating that while the fibroblasts are largely deactivated, they do not fully revert to a homeostatic baseline expression profile. This serves to highlight how sophisticated the zebrafish post-injury fibrotic response is and though there is resolution of the scar in the zebrafish, there remains a careful control of the extracellular environment for months after the fact.

## 8. Discussion

Human therapeutic cardiac regeneration is a long sought-after goal, and the heart is an area of rapid expansion in zebrafish disease modelling, though the phylogenetic distance between zebrafish and mammals means that important questions remain to be answered about the translatability of these studies. This review has highlighted the myriad benefits of zebrafish as a model. The extensive repertoire of cardiac injuries available for study in the zebrafish invites elegant solutions to a number of difficulties posed by other animal models: live imaging capabilities have provided unprecedented levels of *in vivo* data on the injury response mechanisms and cell–cell interactions therein^37^ and drug screens with *N*-numbers well into the thousands have enabled low-cost, high-throughput drug discovery.^27^ The Zebrafish Model Organism Database (ZFIN) (https://zfin.org) is available to researchers as a central repository for genetic and phenotypic data on zebrafish models of human disease.^103,104^ This encourages open science and high reproducibility. Open-source online databases—like ZFIN—have also aided precision genome editing. In this regard, CRISPR/Cas9 has been one of the most important technical advances for zebrafish disease modelling. A major caveat that complicates genetic studies in the zebrafish is the duplication of many genes, making the creation of knockdown strains difficult, which can confound forward genetic approaches due to potential compensatory mechanisms. Advances in RNA and proteomic sequencing at the single-cell level will help us to gain a much fuller picture of the transcriptional and translational profile of the zebrafish heart at key timepoints after injury. This is supported by the constantly expanding database of regulatory miRNAs involved in zebrafish cardiac regeneration, with insight into the differences in expression levels of conserved targets in non-regenerative mammals after injury.^64,66^ This review has discussed the cohesive inter-system response of the zebrafish heart to injury, though there remains plenty to be discovered and the translational benefits of these findings remain to be fully proven. A greater degree of collaboration between groups focusing on different cell types will provide a better understanding of inter-tissue interactions during regeneration. With the gaining popularity of zebrafish models, researchers await an increase in the number of reagents—such as antibodies—suited to zebrafish tissues, the current paucity of which can impede in-depth molecular investigations. Attempts at augmenting human recovery have thus far tended to rely on cardiomyocyte stimulation alone, but these single-system strategies have inevitably resulted in sub-optimal repair.^105,106^ Success in human healing will likely depend on a total temporal understanding of the transcriptional, translational, and post-translational regulation of the myocardium, epicardium, endocardium, vasculature, lymphatics, and immune responses taking into account changes to the extracellular environment. The zebrafish has made significant contributions to the field of cardiovascular science and continues to impress upon us the complexity of a robust regenerative programme. The freedoms afforded in zebrafish research are unparalleled in other models; the future for this regenerative powerhouse is bright.

**Conflict of interest:** none declared.

## Funding

This work was supported by the Wellcome Trust [grant number 108906/B/15/Z to K.M.R.S.]; and the British Heart Foundation [grant numbers FS/19/55/34890 to S.L.W., CH/11/2/28733, RG/14/3/30706 and RM/17/3/33381 to A.H.B., CH/11/1/28798 to P.R.R., and FS/16/4/31831 to M.B.].

## Notes

This paper was handled by consulting editor Jeremy D. Pearson.
